# The Influence of Disease Perceptions on the Participation of Melanoma Patients and their Partners in Skin Self-Examination Education

**DOI:** 10.4172/2161-0711.1000242

**Published:** 2013-11-01

**Authors:** Gaber Rikki, Hultgren Brittney, Stapleton Jerod, A Mallett Kimberly, Turrisi Rob, Hernandez Claudia, Bilmoria Karl, D Wayne Jeffrey, C Martini Mary, K Robinson June

**Affiliations:** Department of Dermatology, Northwestern University Feinberg School of Medicine, Chicago, IL 60611, USA

**Keywords:** Melanoma, Early detection, Disease perception, Partner assistance

## Abstract

By examining differences between patients who enroll or decline to enroll in a partner-based study, future research can benefit and adapt recruitment strategies to reduce sampling biases. This study examined differences between melanoma patients’ who either declined or enrolled in an intervention aimed at increasing skin self-examination (SSE) with partner assistance. Specifically, differences were assessed for gender, age, perception of likelihood of getting another melanoma, benefits of early detection, and severity of the disease. Additionally, reasons for declining were examined. Among 368 melanoma patients interviewed during their appointment with the treating physician, 187 enrolled in the study and 181 declined to participate. No significant age or gender differences between enrolled and declined patients were observed. However, enrolled participants had significantly higher reports on the likelihood of getting another melanoma, severity of melanoma, and early detection as being beneficial (p<0.001). Among those declining to participate, males reported being “too busy and can’t make follow-up appointments” whereas females reported their “partner won’t assist”. Results indicate perceptions of the benefits of early detection, the severity of melanoma, and patients’ increased risk of developing a melanoma may have influenced patients’ decision to participate. Future studies may benefit by highlighting these topics in order to motivate more patients to participant in partner studies.

## Introduction

Social support regarding health-related behaviors has been shown to improve health outcomes, such as having better overall health and living longer, with “socially-integrated individuals” than those who are more isolated [1]. Direct social control, the collection of “request, reminders, rewards or threats” directed at a social partner that influences his or her behavior, can be an effective tool in promoting healthier behaviors for both people in the partnership [2]. The potential benefit of having a partner to assist in such matters is of great relevance to research concerning disease-prevention education to enable early detection of melanoma since such research is often focused on and reliant upon the dyadic relationship of a patient and partner. Robinson et al. found that the supportive, active role of a partner in skin self-examination (SSE) is essential to increasing the self-efficacy of a person with a history of melanoma in the practice of early detection of cancer. In addition, partner-assistance in SSE allows a trained observer to view places on the body that can be difficult for an individual to view (i.e., the back, the scalp) [3]. Our prior research determined that males more readily perceive border irregularity and females perceived color variability in pigmented lesions [4]. Since both border and color are essential to detect a change in a pigmented lesion, men and women working together as a pair enhances the detection of change. Females, who more readily perform SSE and participate in health promotion research than men [5], may motivate their spouses to participate in partner-assisted skin examination research.

Recruitment for partner studies has often been a challenge. Bowen et al. found that the most frequent reason for family members of a melanoma patient declining to participate was being too busy or not having enough time [6]. Without partners willing to participate, research looking at the benefits of supportive relationships for cancer detection suffers. It is important for the success of future research projects involving partners to understand why eligible partnerships decline to participate in studies, so as to find ways to motivate people to participate. The theoretical model guiding our present research is grounded in the Health Belief Model, which has been found to be a useful framework in exploring the role of knowledge and perceptions on personal responsibility [7]. The goal of this study is to examine the influences of attitudes about risk of developing the disease, the severity of the disease and the benefit of early detection on melanoma patients and their partners’ participation in educational research to enhance early detection of melanoma. In addition, given that gender is important in adoption of SSE practices, we examined the data for differences in recruitment and disease perceptions by patients’ gender.

## Methods

The study was conducted in the outpatient clinics of the Northwestern Memorial Faculty Foundation in Chicago, Illinois where researchers had access to patients, who had previously been diagnosed with melanoma. Prior to their appointments at the clinic, melanoma patients with Stage 0 to IIB disease were identified by review of electronic medical records. The inclusion criteria were having a history of Stage 0 to IIB melanoma and being at least 6 weeks after surgical treatment of melanoma, able to see to read a newspaper, fluent in English, age 21–80, and had significant other person (spouse, partner, close relative) who was willing to participate in the research. Exclusion criteria were a history of Stage III or greater melanoma; ocular, genital, and oral melanoma; person overburdened with co-morbid disease, unable to see to read a newspaper, unable to speak English, unable to participate in conversation at a sixth grade language level due to cognitive impairment, and did not have a significant other person (spouse, partner, close relative) who was willing to participate in the research.

At the visit with their dermatologists and surgical oncologists, research coordinators interviewed eligible patients. The coordinator asked the patient to respond to questions regarding their perceptions of their likelihood of getting another melanoma, severity of melanoma and benefit of early detection using a scale from 1–7 (Table 1). The patients were asked about their interest in participating in a study where they learn with a skin-check partner how to examine their skin and detect abnormal features in pigmented lesions for possible melanoma. The patient then chose to enroll or decline to participate. The Institutional Review Board of Northwestern University approved the study.

If a patient declined to participate, the patient was asked to provide a reason. After the first 30 responses were obtained, open ended responses were grouped (Table 2). Subsequent patients (N=158) were asked to select the one response that most closely corresponded to their reason for declining. The data collected included self-reported age and gender of the patient.

### Data analyses plan

First, enrolled participants and those who declined were compared on age and gender using a t-test and a chi-square, respectively. Next, participants and those who declined were compared on the following risk perceptions with independent t-tests: 1) the risk of developing another melanoma, 2) the severity of melanoma, and 3) the benefit of early detection of melanoma. Similarly, gender differences on the three risk perceptions using t-tests within an enrollment category were also examined (e.g., for those who declined). Finally, gender differences for the reasons cited for declining to participate were compared using chi-square tests.

## Results

Among 368 melanoma patients, 181 declined and 187 enrolled (Table 2). There was no significant difference between participants and those who declined on age (t=-.226, *p*>.05) and gender (χ^2^=1.14, p>.05; Declined: Males *n*=116, 52%, Females *n*=147, 46%).

Examination of the means in Table 3 revealed that enrolled participants reported a higher likelihood of getting another melanoma (t=−11.53, *p*<.001), perceived another melanoma as a more serious disease (t=−13.44, *p*<.001), and reported early detection as being more beneficial (t=−16.05, *p*<.001) relative to those who declined. When examining gender differences for those individuals who declined, females reported a higher likelihood of getting another melanoma (t=4.66 *p*<.001), perceived another melanoma as a more serious disease (t=4.46, *p*<.001), and reported early detection as being more beneficial (t=5.17, *p*<.001) relative to males. No significant gender differences were observed for similar analyses examining individuals who enrolled (all *p*’s > 0.05).

(Table 4) Finally, when examining gender differences on reasons cited for declining, males reported being “too busy and can’t make follow-up appointments” whereas females reported that their “partner won’t assist”.

## Discussion

A significant portion of melanoma patients who declined to participate in the study, particularly males, had lower perceived risk of developing a future melanoma and may therefore have had limited motivation to participate in early detection activities. Females who declined often gave “partner won’t assist” as a reason for choosing not to participate in the study. These findings identified a potential concern of overrepresentation of supportive couples in the current study. Supportive couple overrepresentation has been seen in other research, such as in partner studies of breast cancer [8].

Competing priorities and low participation rates by men with melanoma can have skewing effects on the research that is collected in partner studies. The attitudes of men in this study regarding the seriousness of melanoma, their likelihood of getting another melanoma and the potential benefit of early detection may have influenced participation of the patient-partner pair in the melanoma educational study. Thus, it was important to provide a variety of recruitment messages that appealed to couples with varying levels of supportiveness in their relationship. By only having supportive partners enrolled into this study, a sample bias would have been introduced. For example, the results of an educational study could have shown a great benefit to the couples, but perhaps it was mainly due to the enrollment of exceptionally supportive couples, not representing the spectrum of the population of partners. Thus, it was crucial to motivate those partners who may have declined to participate to join their partners in the research study. Enrolling less supportive pairs in the study and determining the efficacy of the SSE educational intervention among a variety of relationship patterns will enhance the potential reach of the program [9].

There is a plethora of research looking at motivating both individuals and partners to participate in research studies, and many strategies have proven effective in increasing recruitment. Recruitment scripts aimed solely at male participation has helped influence recruitment for couple’s studies [8]. Such strategies, like “powerism”, where males were told they were in full control of their participation in the research (i.e. where/when to meet, withdrawal from study, etc.) have been a powerful, positive influence on their participation [10]. In this early detection of melanoma partner study, the partner was needed to perform a visual inspection of the patient; therefore, the decision for the day and time of the appointment was often placed with the male partner. Another recruitment strategy used in this study was offering Saturday and early evening appointments to limit time away from work. If the couple chose to come on Saturday, they often brought the children. Delivery of the recruitment message by the physician trusted by the patient may have helped to encourage participation. In future studies, the doctor and/or research coordinator could use strategies similar to the social control often present in partnerships to request participation by reminding patients about the importance of contributing to research to help others in their family and how the pair can benefit from early detection education, and from having a partner to help in maintaining one’s health.

There were some minor limitations to the study that should be noted. First, the study was conducted at one site during a period of economic recession, where the socioeconomic status of most patients is on average much higher than the general population. This may have an effect on whether the partner perceived he or she had the time to participate in the research. The study was limited in its scope to patients with a history of melanoma Stage 0 to IIB and their skin check partners, but the strategies of encouraging early detection may still be relevant to other studies examining disease prevention.

## Conclusions

When approached to participate in a research study, patients’ attitudes about the severity of the disease, their risk of developing the disease and the possible benefit of the research should be addressed directly in a positive manner in order to enhance subject activation and enrollment. The decision of the patient and/or the couple about enrolling in the research study may be influenced by brief messages enhancing the personal relevance of these three items.

## Figures and Tables

**Table 1:** Survey Questions Asked to Eligible Melanoma Patients.

Question	Scaled Possible Answers
1. In your opinion, what is the likelihood of you getting another melanoma?	Very unlikely (1) to very likely (7)
2. How beneficial is early detection in relation to successfully treating melanoma?	Not at all beneficial (1) to very beneficial (7)
3. How serious of a disease is melanoma?	Not at all serious (1) to very serious (7)

**Table 2:** Influence of Gender and Age on Enrolling in a Melanoma Study (n=368).

Variable	Declined (n=181)	Enrolled (n= 187)
Gender		
Males	100	92
Females	81	95
Age (mean, STD)	54.43 (13.60)	54.65 (16.00)
Age Groups		
18–24	0	3
25–40	31	29
41–65	105	114
66–80	45	41

**Table 3:** Gender and Age Differences in Attitudes about Melanoma.

Variable	Declined		Enrolled	
	Mean	St. D	Mean	St. D
1) What is the likelihood of you getting another melanoma?	3.64	1.928	5.56	1.356
Gender				
Male	2.98	1.764	5.50	1.363
Female	4.26	1.916	5.61	1.355
2) How serious of a disease is melanoma?	4.82	1.862	6.74	0.612
Gender				
Male	4.29	1.748	6.77	.494
Female	5.47	1.789	6.72	.710
3) How beneficial is early detection in relation to successfully treating melanoma?	4.51	1.996	6.93	0.395
Gender				
Male	3.80	1.853	6.92	.339
Female	5.33	1.884	6.93	.443

**Table 4:** Male vs. Female Reasons for Declining. N=181 (8.2% had an open ended question and 91.8% had a forced choice).

Reason	Male	Female
Can’t make follow-up appointments	74	24
Partner will not assist	3	31
Partner cannot assist	2	8
Only want doctor to check my skin	14	9
Unspecified	1	2
Doesn’t live in the area full time	6	7
Total	100	81
